# Supplementary material for: Isotopic and Chemical Assessment of the Dynamics of Methane Sources and Microbial Cycling during Early Development of an Oil Sands Pit Lake
Source: Microorganisms. 2021 Dec 3;9(12):2509. doi: 10.3390/microorganisms9122509 (PMC8703832; doi:10.3390/microorganisms9122509)

Isotopic and chemical assessment of the dynamics of methane sources and microbial cycling during early development of an oil sands pit lake

Slater G.F.[1], Goad, C.A.[1], Lindsay, M.B.J.[2], Mumford, K.G.[3], Colenbrander Nelson T.E.[1], Brady A.L.[1], Jessen, G.L.[*], Warren, L.A.[1,4].

Supporting Information

1 Calculations of Methane Mass Loading from Bubble Dissolution

The mass loading related to dissolution from bubbles during transport was modelled using the model of Leifer and Patro (2002). This model simulates the mass transfer of multiple gas components between the water and a single rising gas bubble. This can result in either bubble dissolution or bubble expansion depending on rates of mass transfer of the various components in and out of the bubble, which depend on the depth, size and velocity of the bubble as well as the concentration of the components in the gas and water phases. The bubble is assumed to be spherical and described by the ideal gas law, such that its radius is:

$$r = \left(\frac{3n_gRT}{4\pi P_g}\right)^{1/3} \tag{S.1}$$

where r is the radius of the bubble, n_g is the total number of moles in the gas phase, R is the ideal gas constant, T is the absolute temperature, and P_g is the total gas pressure in the bubble. The total gas pressure is dictated by the surrounding water pressure, which is assumed to be hydrostatic, and the capillary pressure associated with the gas-water interface:

$$P_g = P_{atm} + \rho_w gz + \frac{2\sigma}{r} \tag{S.2}$$

where P_{atm} is the atmospheric pressure, ρ_w is the water density, g is acceleration due to gravity, z is the depth of water below the lake surface, and σ is the gas-water interfacial tension. The depth of the bubble decreases as it rises:

$$\frac{dz}{dt} = -v \tag{S.3}$$

where v is the bubble velocity. Liefer and Patro (2002) reviewed several expressions for estimating v. In this study, their expression for dirty bubbles (i.e., those with a gas-water interface contaminated with surfactant) with 0.6 mm < r < 10 mm was used:

$$v = 276r - 1648r^2 + 4882r^3 - 7429r^4 + 5618r^5 - 1670r^6 \tag{S.4}$$

where r is expressed in cm and v is in cm/s. For the conditions investigated in this study, particularly an initial bubble radius of 1-5 mm, Equation S4 predicted bubble velocities of 0.15-0.23 m/s, which is in good agreement with bubble velocities measured by sonar of 0.19-0.26 m/s. The mass transfer of each component (i) across the bubble interface is described by diffusion through a thin stagnant film, combined with Henry's Law and Dalton's Law and assuming an interfacial area for a spherical bubble (Leifer and Patro, 2002):

$$\frac{\partial n_i}{\partial t} = k_i 2\pi r^2 \left(C_{i\infty} - \frac{n_i P_g}{n_g H_i} \right) \qquad \text{(S.5)}$$

where n_i is the number of moles, k_i is the mass transfer coefficient, $C_{i\infty}$ is the dissolved concentration in the water column away from the bubble interface, and H_i is the Henry's Law coefficient, all for component i. As was done for the bubble velocity, Liefer and Patro (2002) reviewed several expressions for estimating k_i. In this study, and consistent with assumptions for predicting bubble velocity, their expression for large ($r > 0.2$ mm), dirty bubbles (originally presented by Clift et al., 1978) was used:

$$k_i = 0.42 g^{0.3} \left(\frac{D_i \rho_w}{\mu_w} \right)^{2/3} \left(\frac{\mu_w}{\rho_w} \right)^{0.4} r^{-0.1} \qquad \text{(S.6)}$$

where g is acceleration by gravity in cm/s^2, μ_w is the dynamic viscosity of water in g/cm·s, ρ_w is the water density in g/cm^3, and D_i is the diffusion coefficient in water in cm^2/s, and k_i is expressed in cm/s.

To estimate the mass of methane released into the water column by a rising bubble, Equations S1-S6 were solved using an explicit finite difference approach using the bubble radius, bubble velocity and partial pressures from the previous time step. A time step of 0.1 s was used to reduce numerical error. The approach was verified against the simulations of a 2500 μm radius methane bubble rising in 5 m of water containing dissolved oxygen and nitrogen presented by Liefer and Patro (2002). In this study, the model was implemented assuming bubbles released from the FFT were composed either entirely of methane or 75:25 methane:nitrogen (by volume), and were allowed to exchange mass with a water column that contained dissolved oxygen, nitrogen and methane. Concentrations of oxygen and methane ($C_{i\infty}$ in Equation S5) varied with depth and were based on observations in this study and those reported by Risacher et al. (2018). The dissolved nitrogen concentration was assumed to be in equilibrium with the atmosphere over the entire depth of the water column. The water temperature also varied with depth, and was based on observations in this study. The results of these simulations are sensitive to the initial radius of the bubble released from the FFT, which is unknown. Therefore, initial bubble radii of 1, 3 and 5 mm were simulated.

Equations S1-S6 simulate the rise and mass transfer for a single bubble. However, an estimate of the potential mass loading of methane to BML associated with dissolution from a steady release of bubbles also requires an estimate of the bubbling rate (i.e., number of bubbles per time). This was based on the reported methane flux from the surface of BML, assuming that only bubble transport results in methane release from the surface, as:

$$R_b = \frac{F_{CH_4}}{n_{CH_4}(z=0)} A_{BML} \tag{S.7}$$

where R_b is the estimated bubbling rate, F_{CH_4} is the flux of methane from the surface of BML measured by eddy covariance (Clark, Drewitt et al., 2012), $n_{CH_4}(z = 0)$ is the simulated number of moles of methane in a bubble when it reaches the water surface, and A_{BML} is the surface area of BML. The mass loading over a depth interval z_1 to z_2 is then given by the product of the bubble rate and the mass of methane released from the bubble over that interval:

$$M_b = -R_b \int_{z_1}^{z_2} \frac{\partial n_{CH_4}}{\partial z} \, dz \tag{S.8}$$

where M_b is the mass loading attributed to bubble dissolution.

2 Mass Loading Calculations

An example simulation of bubble rise and mass transfer for an initial bubble radius of 3 mm is shown in Figure S1. The bubble rises at a near-constant velocity (0.19 m/s) through the 10 m-high water column, reaching the water surface in 53 s. The radius increases due to a decrease in water pressure despite the mass transfer of methane out of the bubble, which is partially offset by the mass transfer of oxygen and nitrogen into the bubble.

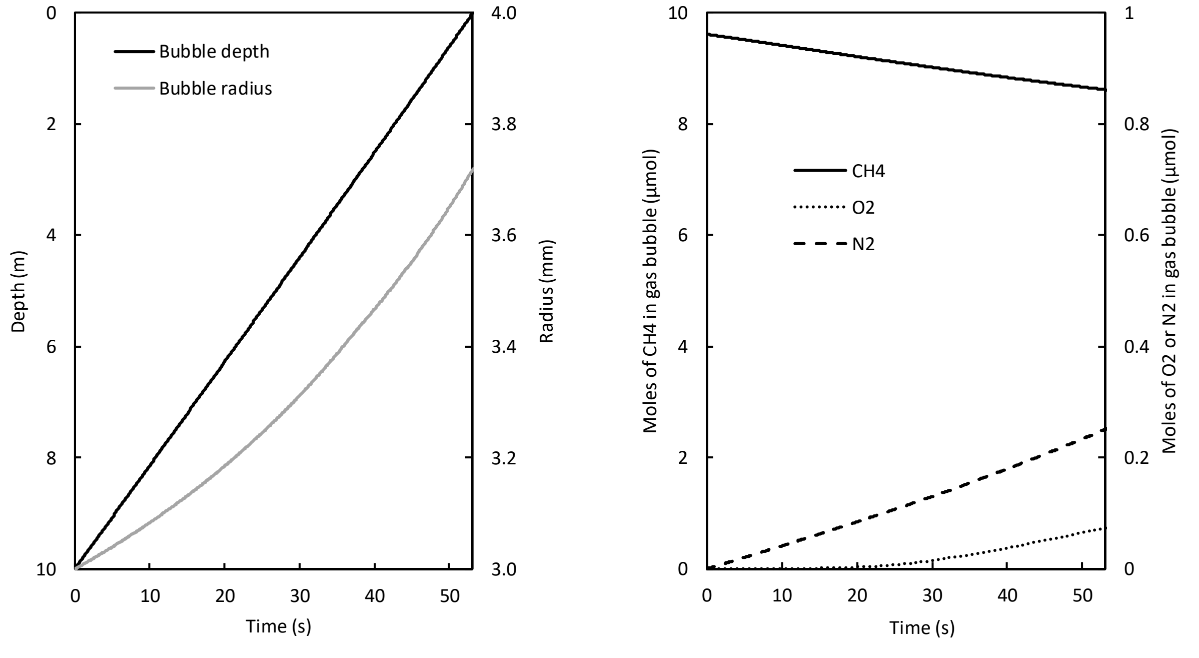

Figure S1. Depth and radius of a bubble rising through a 10 m water column, with an initially radius of 3 mm and an initial composition of 100% methane (left), and the moles of methane, oxygen and nitrogen in the bubble over its 53 s rise time (right).

Equations S7-S8 were used to estimate the mass loading from a steady release of bubbles for three initial bubble radii (1, 3 and 5 mm) and at depth intervals of 0-5 m, 5-10 m and 0-10 m. The estimated bubbling rate was 0.02, 0.12 and 5 bubbles/m^2/min for 100% methane and 0.03, 0.16 and 6 bubbles/m^2/min for 75% methane bubbles with initial radii of 5, 3 and 1 mm, respectively, with higher bubbling rates required for smaller bubbles to match the methane flux measured by eddy covariance. Over all three bubble scenarios and two initial bubble compositions, the mass loading from the bubbles to the water column ranged from 1.8×10^5 to 4.7×10^6 moles/year, with higher mass loading occurring for small initial radii and at deeper depth intervals. This is consistent with smaller gas bubbles having a higher surface area-to-volume ratio and bubbles closer to the FFT having a greater concentration of methane, both of which facilitate faster mass transfer. Importantly, this mass loading occurs throughout the bubble rise (Figure S1), with 53-57% of the methane from the bubbles released to the water column in the 5-10 m depth interval. The degree of contamination of the gas-water interface (i.e., clean or dirty bubbles) is unknown, resulting in uncertainty associated with both the bubble velocity and mass transfer rate coefficient.

3 FFT Methane Isotopic Compositions

Figure S2: Stable isotopic ($\delta^{13}C$, δ^2H) compositions of dissolved methane in BML FFT. Boxes indicate empirical ranges for fermentative and hydrogenotrophic methanogenesis adapted from Scholl et al 1988.

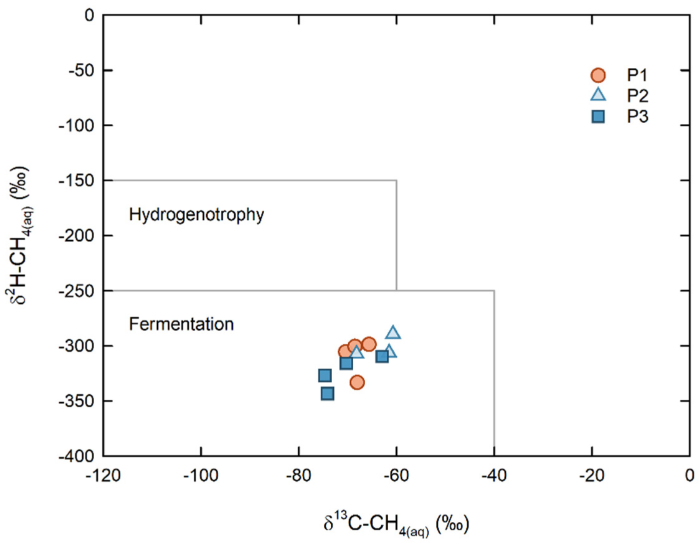

Supplement: Supplementary file 1 [file microorganisms-09-02509-s001.zip › microorganisms-1463821-supplementary.pdf]
